# Towards democratised psychiatry: building metacommunities for inclusive and equitable global mental health

**DOI:** 10.1192/bji.2024.30

**Published:** 2025-02

**Authors:** Yansen Alberth Reba, M. Zaenul Muttaqin, Yovian Yustiko Prasetya

**Affiliations:** 1 Lecturer, Guidance and Counseling Study Program, Cenderawasih University, Jayapura, Papua, Indonesia. E-mail: yansenreba070189@gmail.com; 2 Lecturer, Public Administration Study Program, Cenderawasih University, Jayapura, Papua, Indonesia; 3 Lecturer, Guidance and Counseling Study Program, Pancasakti University Tegal, Tegal, Central Java, Indonesia

**Keywords:** Democratised psychiatry, mental health, technological challenges, public policy, health economics

## Abstract

Through their study, George Ikkos & Nick Bouras reveal that the responsibilities of the psychiatric community are increasingly complex, especially amid the onslaught of globalisation and the confines of neoliberalism. ‘Metacommunity’ in this context refers not only to the history of psychiatrists but their role in strengthening and influencing mental health policies. A number of challenges continue to emerge in the public sphere, highlighting the need for psychiatry to adapt to society's evolving demands for inclusivity, equity and ethical governance. These challenges emphasise the importance of shaping the future of psychiatry that is responsive to the complexities of mental health care and aligned with democratic principles that prioritise transparency and social accountability. We have added several aspects that could complement psychiatrists' future theory and practice, including a more collaborative and evidence-based approach to dealing with increasingly complex mental health issues.

Ikkos & Bouras offer an interesting analysis of the failure of the community psychiatry approach to achieve its original goals in high-income countries such as the USA and UK.^1^ They accurately identify how factors such as neoliberal policies, globalisation and digital technology have drastically altered the societal landscape on which the concept of ‘community’ was originally based.

A historical approach shows that mental health services cannot be separated from underlying structural economic problems. In high-income countries, these policies often ignore socioeconomic inequalities, further exacerbating the mental health conditions of communities.^2^ For example, mental health policies in the USA and UK often focus more on individuals and medical treatment than on understanding the social and economic roots of mental health problems.^3^ This has led to ineffectiveness in addressing broader issues such as poverty, unemployment and social marginalisation.^4^

The updated interpretation of metacommunity proposed by Ikkos & Bouras^1^ provides a useful framework for understanding this transformation and highlights the need for psychiatrists and other mental health providers to adopt socially critical thinking and active engagement in the public sphere.^5^ Globalisation and social media have created ‘new communities’ that differ from the notion of geographically based communities that underlie community psychiatry. These virtual communities, while offering emotional and informational support, are often unable to replace the physical and social support provided by locally based communities – as evidenced by numerous studies on social solidarity, counselling services for female victims of bullying, and trauma healing based on local wisdom in the aftermath of natural disasters.^6–9^

In this context, psychiatrists and mental health service providers in high-income countries need to adopt a more holistic and democratic approach to their practice.^10^ We agree that to address broader structural challenges, such as socioeconomic inequality, we need a ‘democratic psychiatry’ that challenges these root causes and ensures equitable access to quality mental health services.^11^ Democratic psychiatry should involve patients, families and communities in decision-making and challenge policies that reinforce social and economic inequities.^12^

However, in low- and middle-income countries, the challenges of providing adequate mental health services are often more complex and urgent. Therefore, we propose the inclusion of perspectives from low- and middle-income countries that face different challenges in providing mental health services.^13^ For example, in many such countries, mental health services are often underfunded and deprioritised in national health systems. Other challenges include stigma towards mental illness, lack of trained mental health personnel and limited access to care.

## The need for horizontal collaboration in research

Ikkos & Bouras posit that the introduction of the concept of metacommunity was intended to elucidate the transformational significance of changes in the modern political economy, including the rapid advancement of clinical and information technology and its impact on psychiatry. Online communities are markedly distinct from geographical communities, offering novel threats and opportunities. It would be valuable to explore how the metacommunity concept can be applied in different political economy contexts. Given that most of the world's population lives in low- and middle-income countries, it is important to understand how a more inclusive and holistic approach can be applied to improve mental health services across different social and economic contexts. In this regard, perspectives from patient advocacy groups, families and civil society are crucial.^14^ Horizontal collaboration with such stakeholders – and also media organisations – will strengthen efforts to reform mental healthcare systems to be more responsive to people's real needs.^15^

The urgency of policy advocacy and community democratic engagement cannot be ignored.^16^ The psychiatric community needs to build horizontal networks with these stakeholders to amplify their voices in policy decision-making.^17^ Intricate sociocultural stratification can exacerbate disparities in access across various domains, including education, technology and mental healthcare, as for example in the patriarchies intrinsic to certain cultures. Furthermore, in the context of gender, where women are denied the opportunity to pursue education, this will inappropriately restrict the number of female mental health professionals and will have a detrimental impact on policy and clinical services for women who utilise these services. Psychiatrists can engage with diverse stakeholders, including forming alliances with activists from non-clinical backgrounds to surmount technological challenges. This will ensure that mental health policies are more responsive to the needs and social realities faced by the community. The insights gained will enrich further discussions on the role of psychiatry in addressing the evolving socioeconomic and technological challenges of the future. With a more inclusive and collaborative approach, we can create a more equitable and responsive mental health system for all.

## Closing notes

This article critiques the failure of community psychiatry in high-income countries due to shifts in the social landscape caused by neoliberal factors, globalisation and digital technology. The concept of metacommunity is proposed as a new framework emphasising the need for psychiatrists to adopt socially critical thinking and public engagement to meet the needs of new communities formed through globalisation and social media. To tackle the challenges of structural inequality, Ikkos & Bouras^1^ call for ‘democratic psychiatry’ that addresses root problems and ensures access to quality mental health services. However, they are overly focused on wealthy countries, while low- and middle-income nations face different challenges.

Recommendations include expanding the perspective to low- and middle-income countries to explore the application of the metacommunity, incorporating views from patient advocacy and civil society, emphasising the urgency of policy advocacy and community engagement, and strengthening the argument with evidence related to mental health service challenges in low- and middle-income countries and cross-stakeholder collaboration. These recommendations aim to provide inclusive and collaborative solutions to build a more equitable and responsive global mental health system. This also substantiates the necessity for the advent of the metacommunity as a global mental health movement.

## Data Availability

Data availability is not applicable to this article as no new data were created or analysed in this study.
